# Supplementary material for: Comprehensive discovery of subsample gene expression components by information explanation: therapeutic implications in cancer
Source: BMC Med Genomics. 2017 Mar 15;10:12. doi: 10.1186/s12920-017-0245-6 (PMC5351169; doi:10.1186/s12920-017-0245-6)
Supplement: Additional file 1 — Supplementary methods details and text including a description of the Bayes prior and reproducibility across runs. (PDF 7511 kb) [file 12920_2017_245_MOESM1_ESM.pdf]

Supplementary Figures and Text

Shirley Pepke[1] and Greg Ver Steeg[2]

1 University of Southern California, Information Sciences Institute (Marina del Rey, CA, USA);**2** Lyric LLC (South Pasadena, CA, USA)

1. Supplementary Figures

see in separate file

Figure 1:

All heat maps located at:

https://app.box.com/s/r2wxxfj89obz47q4qxrcdpf9lj4czu26/SuppFig1.heats.pdf

see in separate file

Figure 2:

All string networks located at:

https://app.box.com/s/r2wxxfj89obz47q4qxrcdpf9lj4czu26/SuppFig2.stringnetworks.pdf

Consider groups X_A^m and X_B^n (mth group from training A and nth group from training B.
If RBO_extn > $RBO_{threshold}$:

$$\{X_A^m, X_B^n\} \rightarrow \begin{cases} \{X_A^m\} & \text{if } TC_N(X_A^m) > TC(X_B^n) \\ \{X_B^m\} & \text{otherwise} \end{cases}$$

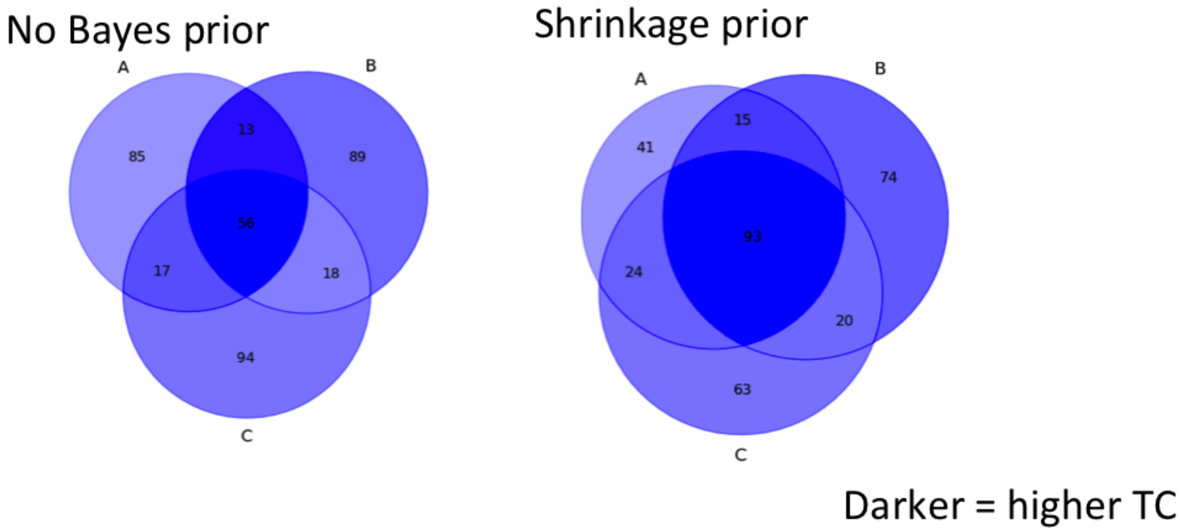

Figure 3:

The rank biased overlap can be used to identify equivalent groups from different runs (to within some tolerance) Run results can be aggregated by declaring groups equal if their RBO exceeds some threshold and then retaining only the group with highest TC from among a set of equivalent groups. Reproducibility between runs was measured using the rank-biased overlap (after excluding very large groups). The Venn diagrams show how utilizing a Bayes shrinkage prior as a smoothing technique increases the fraction of groups that are identified as equivalent according to the RBO measure. In both cases, groups with higher TC or more likely to appear relatively unchanged across runs.

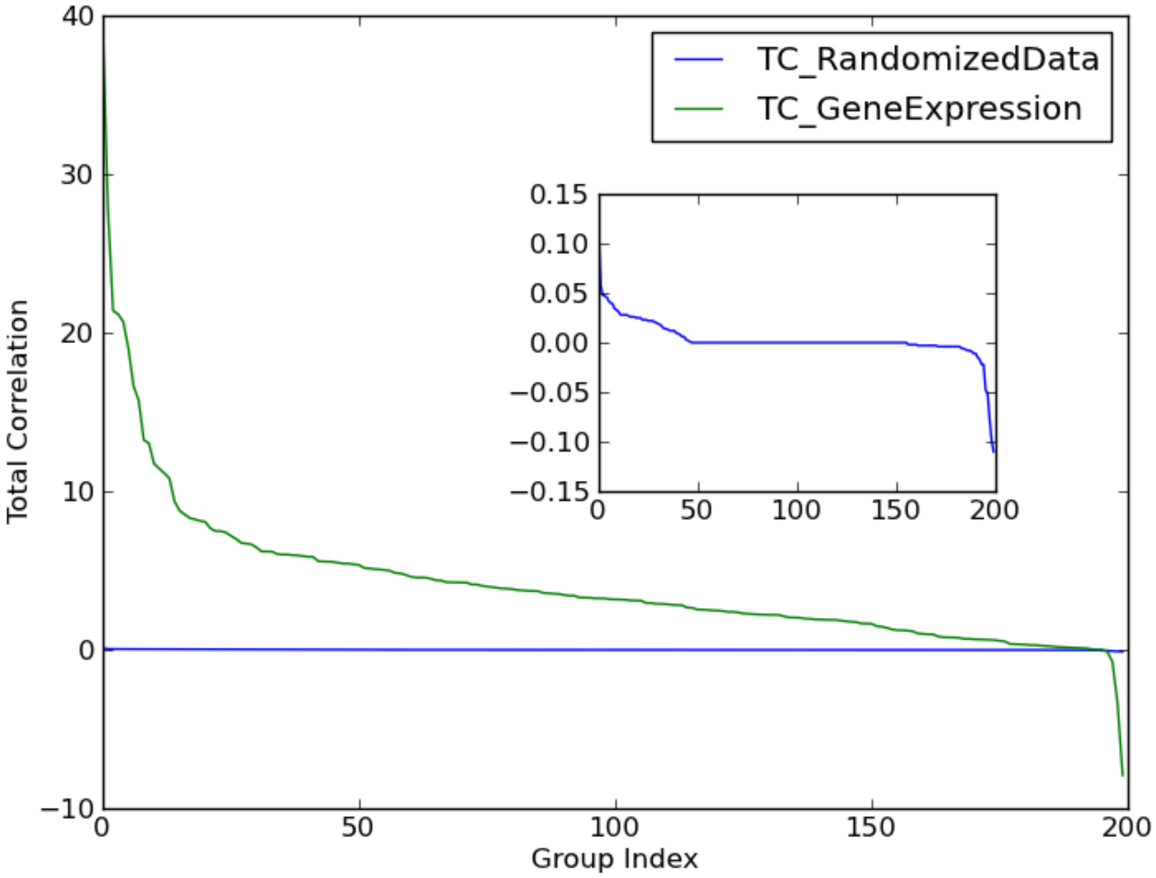

Figure 4:

Randomization test of algorithm. The plot shows typical CorEx group Total Correlation values for the TCGA gene expression matrix and the same matrix with values randomly permuted.

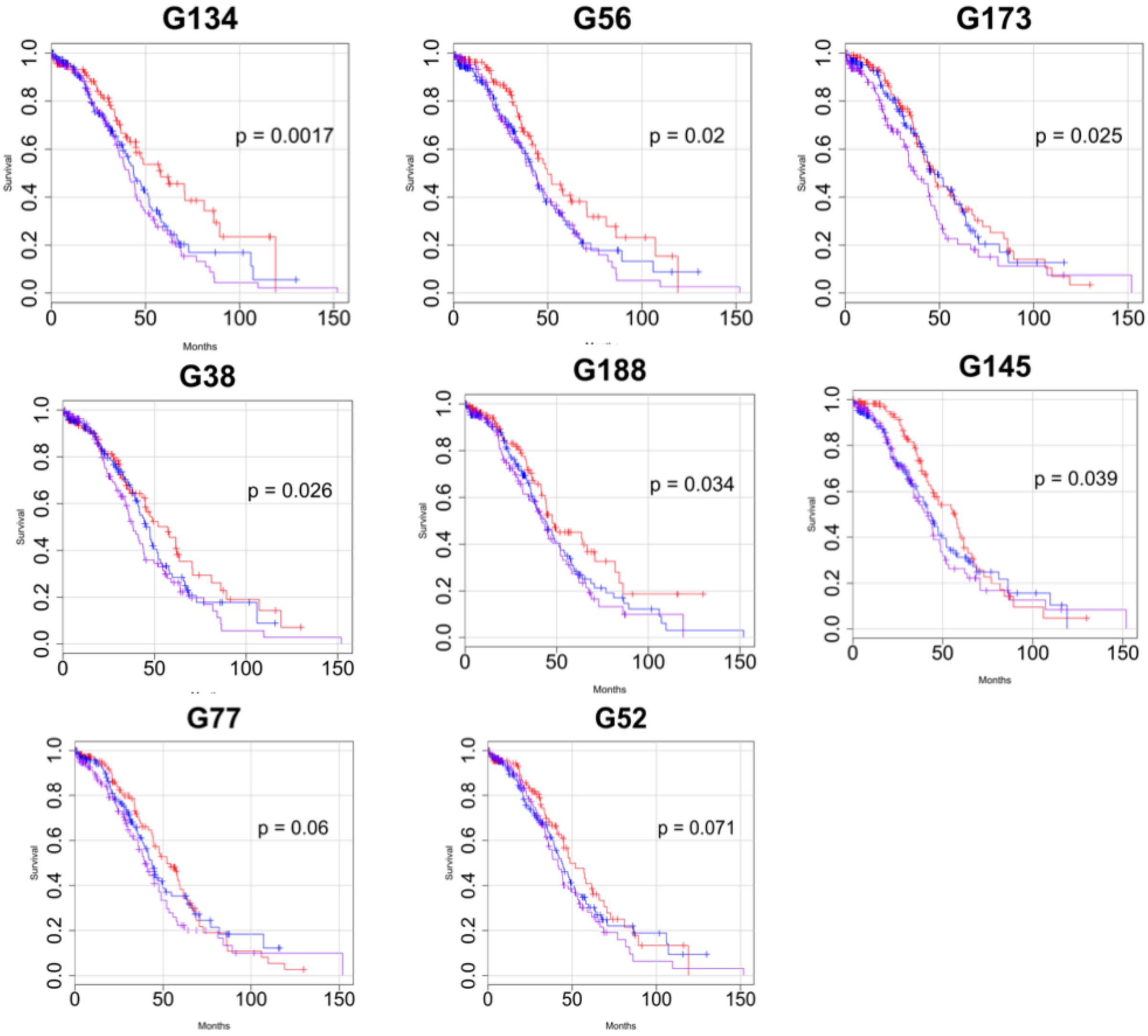

Figure 5: *Supplementary survival curves*

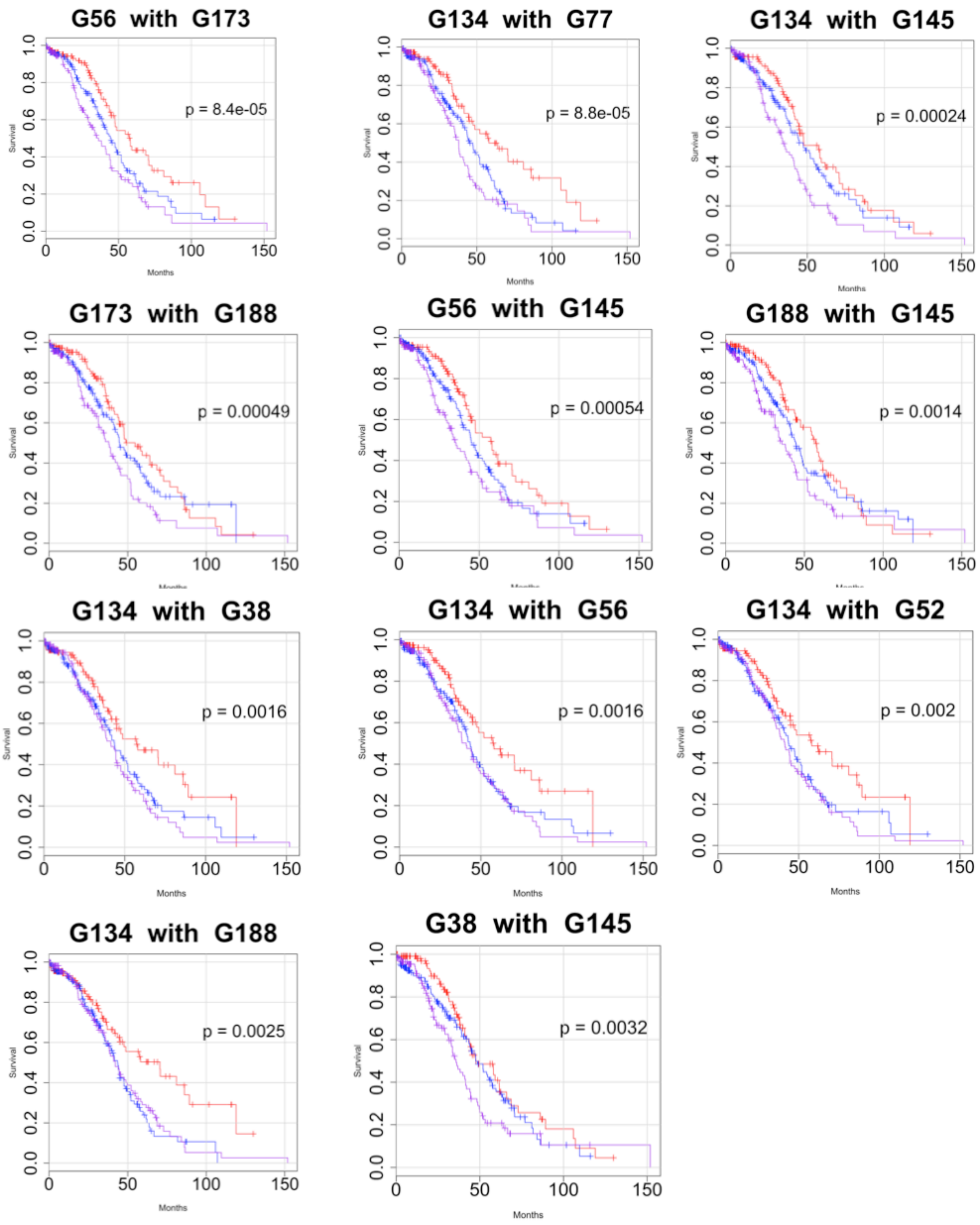
G56 with G173
p = 8.4e-05
G134 with G77
p = 8.8e-05
G134 with G145
p = 0.00024
G173 with G188
p = 0.00049
G56 with G145
p = 0.00054
G188 with G145
p = 0.0014
G134 with G38
p = 0.0016
G134 with G56
p = 0.0016
G134 with G52
p = 0.002
G134 with G188
p = 0.0025
G38 with G145
p = 0.0032
Survival
Months

Figure 6: *Supplementary combination survival curves*

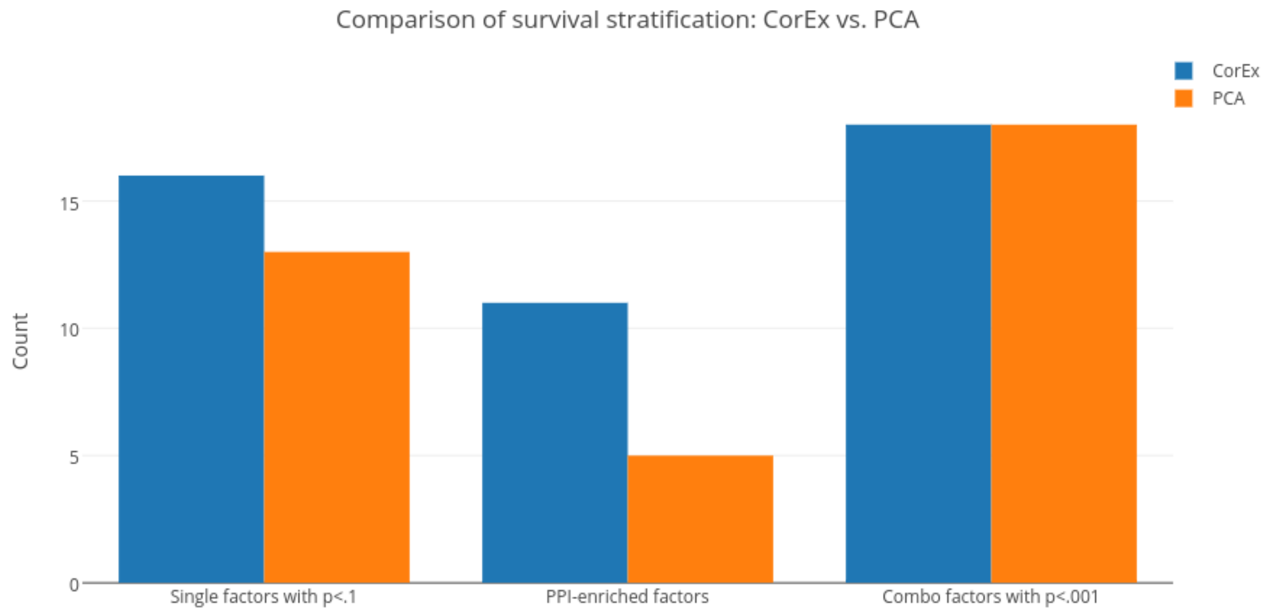

Figure 7:

Comparison of significance of stratifying patients to predict survival. While the PCA and CorEx factors can yield comparable survival associations, the CorEx factors are more enriched for protein-protein interactions. The first PCA component accounts for only 1.6 percent of the total sample variance, while the 13 principal components for which p<.1 account for a total of 5% together.

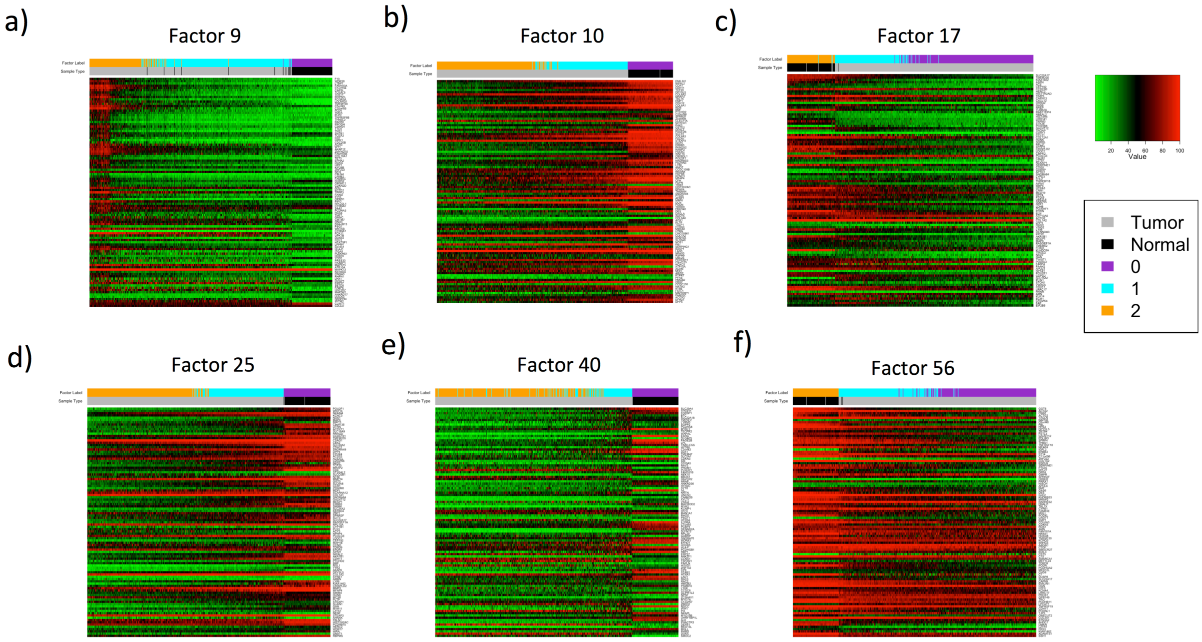

Figure 8:

Heat maps for factors highlighted in Figure 9 in the main text. Expression values are intrasample percentile values for each gene. Factors were learned using the same structure as for the tumors alone.

2. Supplementary Text

Given the variability between runs of CorEx in such a high dimensional space, some way to compare and reconcile the results of multiple runs is needed. We have found that comparison of the gene groupings using the rank-biased overlap (RBO) of **[1]** can be used to draw intuitively satisfying correspondences. The RBO is calculated as a single number that describes the similarity between two ranked lists. It is very flexible and has appealing asymptotic properties. It is possible to usefully aggregate results from various runs by declaring any two groups that exceed a threshold RBO to be equivalent. When this is the case, the group with greater TC is retained.

3. Supplementary Methods

3.1. Estimation of parameters

3.1.1. Bayesian shrinkage estimates for the marginals

The remaining details of the learning scheme concern the estimation of parameters in terms of our samples of data x, and the current estimate of probabilistic labels, $p(Y_j = y_j|X = x)$. First of all, as we mentioned, $p(X_i = x_i|Y_j = c)$ is estimated as a normal with mean $\mu_{i,j,c}$ and variance, $\sigma_{i,j,c}$. Let x^l denote the l-th sample of data. Then an empirical estimate for μ, would be the following.

$$\mu_{i,j,c}^{ML} = (\sum_l p(Y_j = c|X = x^l)x_i^l)/(\sum_l p(Y_j = c|X = x^l)) \tag{1}$$

We could simply use this estimate and the corresponding one for variance. However, if there are a small number of samples with label $Y_j = c$, this could become quite noisy. Instead, we consider a Bayesian estimate based on James-Stein type shrinkage estimators **[2,3]** . We take as our Bayesian prior the hypothesis that the mean is $\mu_i{}^0 = 1/N\sum_i x_i{}^l$.

A Bayesian estimate for the mean of a normal distribution has a simple form.

$$\mu_{i,j,c} = \lambda_{i,j,c}\mu_{i,j,c}^{ML} + (1 - \lambda_{i,j,c})\mu_i^0 \tag{2}$$

The main question is how to set the value of the ``shrinkage parameter", λ. The idea behind shrinkage estimators is to analytically estimate the value for λ to be the one with the minimum risk. We derive our estimate here since the setting differs slightly from previous attempts **[2,3]** .

For simplicity, we drop the subscripts and assume the true distribution has mean, μ, the prior is μ^0, and the empirical estimate is $\hat{\mu}$, and that the standard deviation is fixed and known σ. Let $p(x; \mu, \sigma)$ be a normal distribution parametrized by μ, σ. The risk, R is defined as the KL divergence between the true distribution and the estimated one.

$$R = \mathbb{E}[D(p(x; \mu, \sigma)||p(x; (1 - \lambda)\mu_0 + \lambda\hat{\mu}, \sigma)] \tag{3}$$

We set λ to be the one that minimizes the risk by taking the derivative and solving as usual. It turns out that this leads to the following expression.

$$\lambda = \frac{(\hat{\mu} - \mu_0)^2}{(\hat{\mu} - \mu_0)^2 + z^2} \tag{4}$$

We have $z^2 = \sigma^2/N$ and we recognize this is just the standard error of the mean. Rather than estimate that, we use a shuffle test to estimate how far we expect $\hat{\mu}$ to be from μ_0 under the null hypothesis, and we call this $\hat{z}$. For some random permutation of the samples, π, we have the following.

$$\mu_{i,j,c}^{\text{shuffle}} = (\sum_l p(Y_j = c|X = x^l)x_i^{\pi(l)})/(\sum_l p(Y_j = c|X = x^l)) \tag{5}$$

Then we take an expectation over several shuffles, $\hat{z}^2_{i,j,c} = \mathbb{E}_\pi[(\mu^{\text{shuffle}}_{i,j,c} - \mu^0_i)^2]$. For ``agressive" shrinkage, instead of shuffle x_i^l, we sample with replacement from the empirical distribution.

3.1.2. Setting the weights

The form of the weights, $a_{i,j}$ are directly determined by the optimization which requires that $a_{i,j} = I(X_i; Y_j | Y_1, \ldots, Y_{j-1}) / I(X_i; Y_j)$, for some ordering of the Y_j. We use a more tractable estimate for this weight **[4]** .

For each sample, x^l, we first calculate the most likely label for each latent factor, $\bar{y}^l_j = \arg\max_y p(Y_j = y | X = x^l)$. We define the prediction of Y_j based on X_i for sample l as $P_{i,j,l} = \arg\max_y p(X_i = x^l_i | Y_j = y) / p(X_i = x^l_i)$. We define, $C_{i,j,l} = 1$ if $P_{i,j,l} = \bar{y}^l_j$ and 0 otherwise. $C_{i,j,l}$ shows whether X_i correctly predicts Y_j for sample l. Next, for each i, we sort j's in decreasing order of $C_{i,j} \equiv \sum_l C_{i,j,l}$. Then we set $a_{i,j}$ to be the fraction of samples for which $C_{i,j,l} = 1$ and $C_{i,k,l} = 0, \forall k < j$. In other words, it is the fraction of times that X_i correctly and uniquely predicted Y_j.

3.1.3. Training higher layers of the hierarchy

At the first layer of the hierarchy, the input variables are continuous and are latent factors are discrete. The corresponding training procedure was described in the main text. Now for each patient, *l*, and for each latent factor, Y_j , we have a distribution $p(Y_j = c|X^l)$. We construct a new data matrix consisting of the most probable value for each latent factor and each patient as $Y_j^l = \arg\max_c p(Y_j = c|X^l)$. This data matrix will now be treated as the input data, *X*, and used to train a new CorEx representation at the next layer. Note, however, that at this next layer, both the inputs and the latent factors are discrete. This leads to a simplification of the update equations presented in the main text. In particular, the marginal distribution, $p(x_i|y_j)$, was previously parametrized as a normal distribution. Now, however, we can directly estimate this marginal from the contingency table of counts of different discrete events.

$$p(X_i = c|Y_j = d) = \frac{1}{N}\sum_{l=1}^{N} p(Y_j = d|X = x^l)\delta_{x_i^l,c}/p(Y_j = d) \tag{6}$$

The symbol, δ, represents the discrete delta function and is 1 if its subscripts match and 0 otherwise. The update equation for $p(Y = y|X = x)$ and the other details of the optimization are unchanged.

3.1.4. PCA Survival Analysis

Principal component coordinates were used analogously to Corex continuous factor labels. The sample population was stratified approximately into thirds relative to the coordinate values for each principal component. All survival analyses (e.g. for Supplementary Figure 7) then proceeded as was done for the CorEx factors.

4. Bibliography

1.

Webber W, Moffat A, Zobel J (2010) A similarity measure for indefinite rankings. ACM Transactions on Information Systems (TOIS) 28: 20–38. Available: http://portal.acm.org/citation.cfm?doid=1852102.1852106.

2.

Hausser J, Strimmer K (2009) Entropy Inference and the James-Stein Estimator, with Application to Nonlinear Gene Association Networks. The Journal of Machine Learning Research 10: 1469–1484. Available: http://dl.acm.org/citation.cfm?id=1577069.1755833.

3.

Schafer J, Strimmer K (2005) A shrinkage approach to large-scale covariance matrix estimation and implications for functional genomics. Statistical applications in genetics and molecular biology 4: Article32. Available: http://www.degruyter.com/view/j/sagmb.2005.4.issue-1/sagmb.2005.4.1.1175/sagmb.2005.4.1.1175.xml.

4.

Ver Steeg G, Galstyan A (2015) Maximally informative hierarchical representations of high-dimensional data. Proceedings of the Sixteenth International Conference on Artificial Intelligence and Statistics AISTATS.
